# THE EFFECT OF CAREGIVING EXPERIENCE ON CARE RECIPIENT OLDER ADULTS’ MORTALITY: A SURVIVAL ANALYSIS

**DOI:** 10.1093/geroni/igz038.796

**Published:** 2019-11-08

**Authors:** Teja Pristavec, Elizabeth A Luth

**Affiliations:** 1 University of Virginia, Biocomplexity Institute and Initiative, Arlington, Virginia, United States; 2 Weill Cornell Medicine, New York, New York, United States

## Abstract

Health and demographic mortality risk factors among older adults are well documented. However, less is known about the dyadic relationship between caregiver characteristics and care recipient mortality outcomes. In a nationally representative sample of older adults, we prospectively explore 1) whether and how having an informal caregiver is associated with care recipient mortality, and 2) among those with caregivers, how caregivers’ experiences of burden and benefits relate to care recipient mortality. We match 6 waves of National Health and Aging Trends Study (2011-2016) with 2011 National Study of Caregivers data. We conduct survival analysis on 7,369 older adults and a subsample of 1,341 older adult-informal caregiver dyads to address our research questions. First, we find that simply having an informal caregiver increases mortality risk by 71% (p<0.001) over the 6-year time period, even when adjusting for key demographic, economic and health factors. Second, we find that older adults whose caregivers perceive burden have a significantly higher mortality risk. This risk is reduced if the caregiver also perceives caregiving benefits. The risk of death is 41% higher for older adults whose caregivers report burden but no benefit compared to those with caregivers who report neither burden nor benefit. Further research should investigate possible reasons why merely having a caregiver increases older adults’ mortality risk. Interventions to increase caregivers’ sense of benefit and reduce their burden may be an effective way of decreasing mortality risk for older adults with declining health and functional ability.

